# A first of its kind: iliacus hernia containing an inflamed appendix

**DOI:** 10.1093/jscr/rjaf362

**Published:** 2025-06-03

**Authors:** Aulon Jerliu, Marwan Alaoudi, Noubar Kevorkian

**Affiliations:** Department of Surgery, University of Connecticut School of Medicine, 263 Farmington Ave, Farmington, CT 06030, United States; Department of Surgery, Hospital of Central Connecticut, 100 Grand Street New Britain, CT 06052, United States; Department of Surgery, Hospital of Central Connecticut, 100 Grand Street New Britain, CT 06052, United States; Department of Surgery, Hospital of Central Connecticut, 100 Grand Street New Britain, CT 06052, United States

**Keywords:** appendicitis, iliacus, hernia, appendix

## Abstract

Iliacus hernias are extremely rare internal hernias with only sporadic reports in the literature. No previous report has documented an inflamed appendix herniating into the iliacus muscle. This report describes such a case, highlighting the diagnostic challenges and the surgical management of this atypical hernia. This case expands the spectrum of internal hernias and emphasizes the importance of advanced imaging and a customized surgical approach when encountering atypical hernia locations. Early recognition and prompt management are critical for this rare hernia type. Further study is needed to establish standardized treatment protocols for internal hernias in uncommon locations.

## Introduction

Hernias involving the vermiform appendix are exceedingly rare, accounting for only ~1% of all hernias [1]. The most frequently encountered types are Amyand’s hernia (appendix within an inguinal hernia) and the even rarer De Garengeot’s hernia (appendix within a femoral hernia) [2, 3]. In Amyand’s hernia, the incidence of finding the appendix in an inguinal sac is ~1% of inguinal hernia cases, but an acutely inflamed appendix is present in only 0.1% [1, 2]. De Garengeot’s hernia comprises ~1% of femoral hernias and is more common in females [2, 3]. Isolated reports also exist of the appendix found in other hernia locations (e.g. incisional, obturator, or Spigelian hernias) [1, 4].

Herniation into the iliopsoas or iliacus muscle is an extremely uncommon phenomenon. To date, only two cases of psoas (iliopsoas) muscle hernia have been documented, both containing preperitoneal fat, discovered incidentally or due to vague symptoms [3]. Goel *et al.* reported a case of a psoas muscle hernia found during endoscopic hernia repair, hypothesizing heavy exertion caused a tear in the psoas muscle [3]. Esmaili *et al*. described an iliopsoas hernia identified during laparoscopic extraperitoneal inguinal hernia repair [5]. Recently, an iliacus muscle hernia containing a perforated ascending colon diverticulum was reported [6]. However, an acutely inflamed appendix herniating into the iliacus muscle has not been previously reported. We present the first such case.

## Case presentation

**Figure 1:**
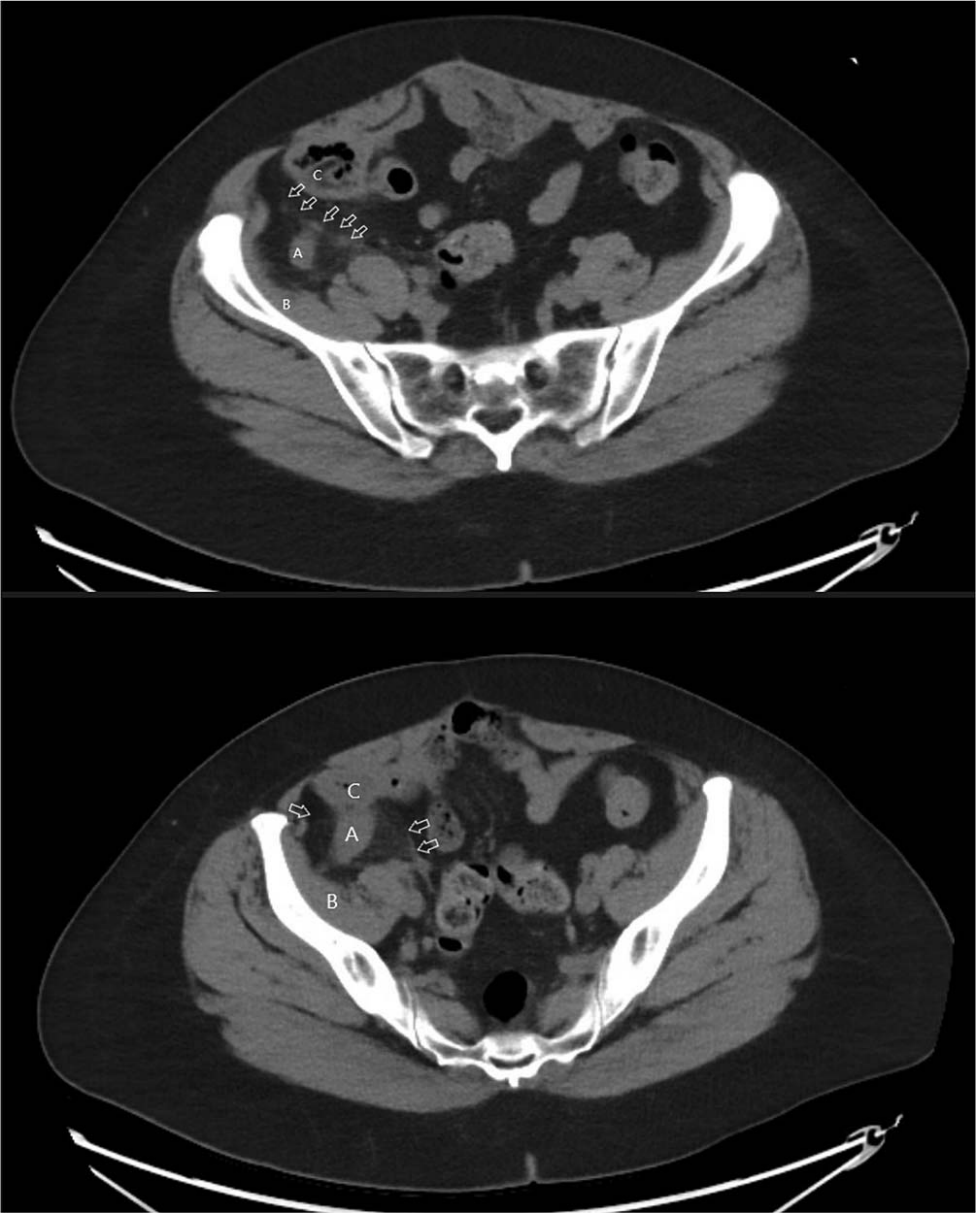
Axial images of CT scan with IV contrast showing appendix within retrocecal position in proximity within iliacus space. (A) Appendix; (B) iliacus muscle; (C) cecum.

**Figure 2:**
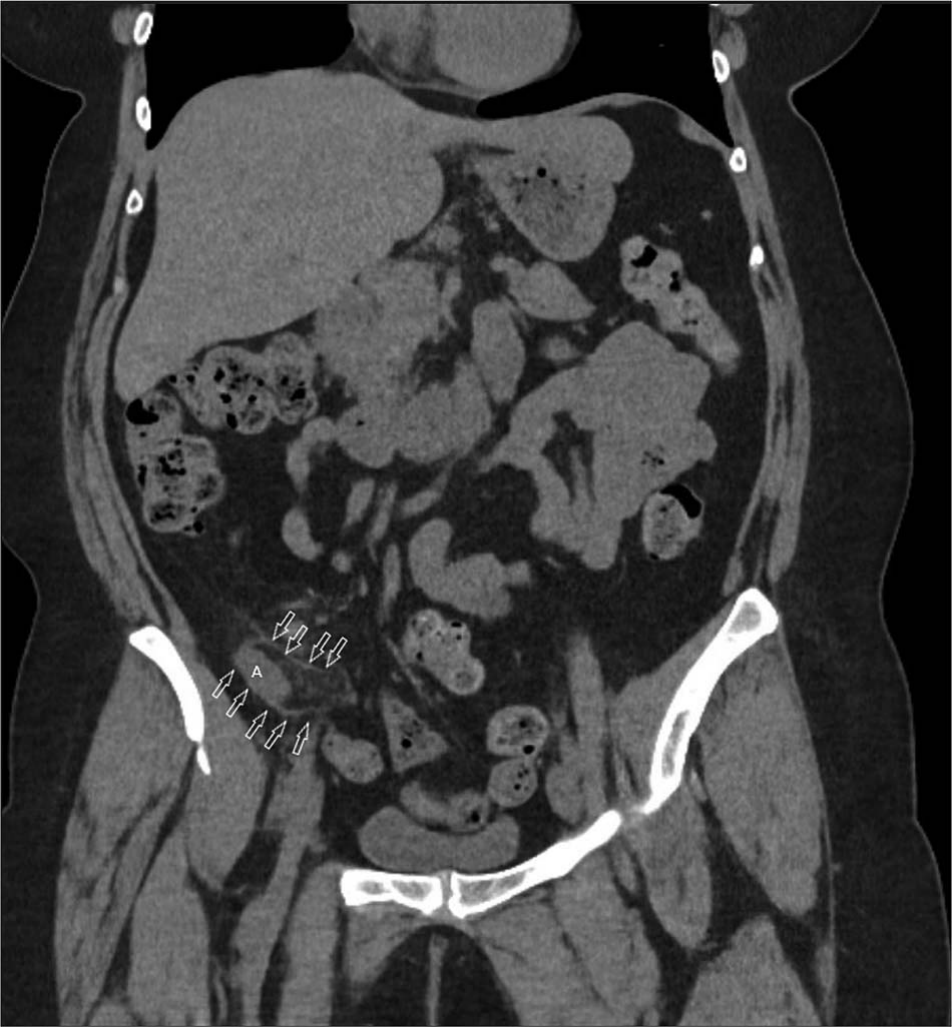
Coronal image CT scan with IV contrast with arrows denoting appendix in proximity to iliacus space. (A) Appendix.

A 58-year-old female with a history of ovarian cancer, status post total abdominal hysterectomy, bilateral salpingo-oophorectomy, and omentectomy, presented with a 1-week history of right lower quadrant and right pelvic abdominal pain. Her labs were unremarkable. A CT scan of the abdomen and pelvis revealed a distended, fluid-filled appendix measuring 1.7 cm in diameter, with associated mesenteric infiltration and several small lymph nodes in the right lower quadrant, consistent with acute appendicitis (Fig. 1). The surgeon’s review of the scan was suspicious of retrocecal appendix (Fig. 2).

**Figure 3:**
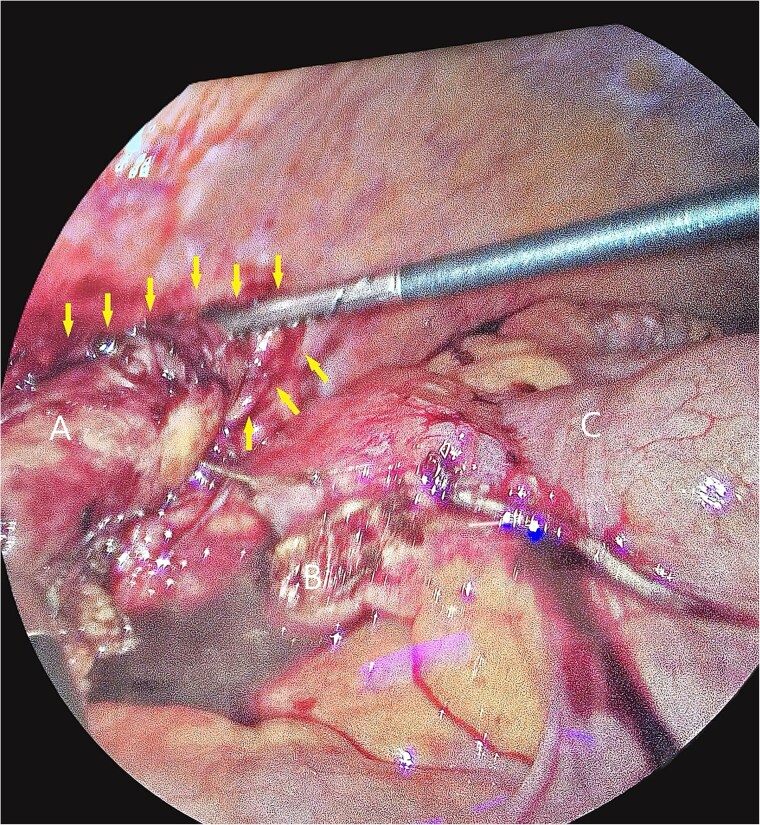
Intra-operative photos of appendix contained within iliacus hernia after appendix was divided. The arrows showcase the hernia with appendix. (A) Appendix, (B) mesoappendix after division, (C) cecum with staple line.

**Figure 4:**
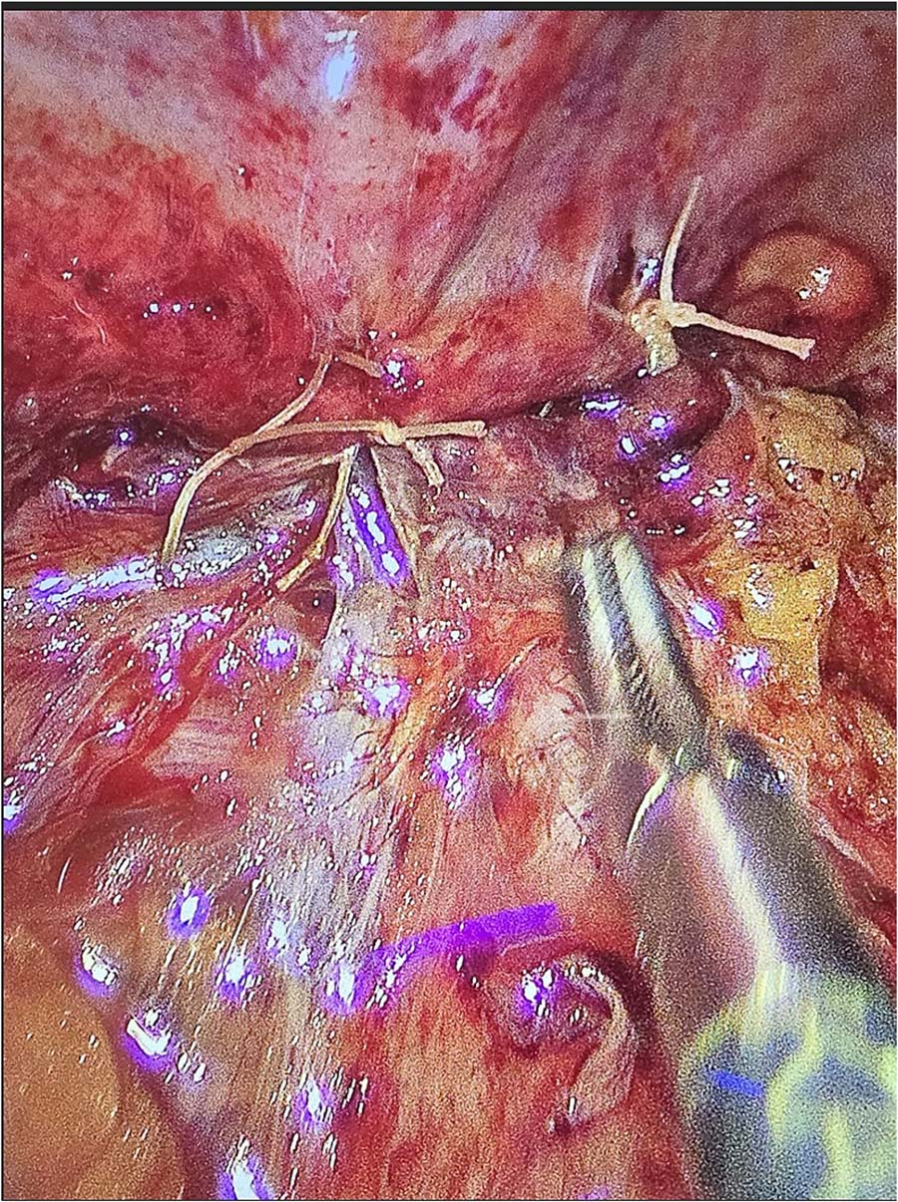
Iliacus hernia defect closed primarily after appendix was removed.

The patient underwent laparoscopic appendectomy with laparoscopic enterolysis. The appendix appeared inflamed, dilated, and gangrenous, with a perforation located 2 cm from the base. Notably, the appendix was not in a retrocecal position as initially suspected but was found to be herniating through a defect in the iliacus muscle (Fig. 3). The appendix was circumferentially freed from the hernia defect, which was identified as tracking cephalad between the iliacus muscle and the iliac bone. The hernia defect measured 2.5 cm and was primarily closed using two figure-of-eight 2–0 Vicryl sutures, leaving a small inferior opening to allow for drainage (Fig. 4). A 19 Fr Blake drain was placed in the right lower quadrant and paracolic gutter.

Postoperatively, the patient’s course was uneventful, the drain was removed, and she was discharged on post operative Day 2.

This case highlights the unusual presentation of an appendiceal herniation through an iliacus muscle defect, a previously unreported entity in the literature. The surgical approach required careful dissection and repair to ensure optimal patient outcomes while addressing both the appendiceal pathology and the hernia defect.

## Discussion

Iliacus muscle hernia is an internal hernia that is extremely rare [3, 5, 6]. Our case uniquely describes an inflamed appendix contained within an iliacus muscle hernia, distinct from more commonly known appendix-containing hernias such as Amyand’s and De Garengeot’s [1–3, 7].

Amyand’s and De Garengeot’s hernias frequently present with nonspecific symptoms like routine incarcerated hernias, often complicating preoperative diagnosis [1, 2]. These hernias emphasize the clinical challenge posed by internal hernias, particularly given their potential for delayed diagnosis and increased morbidity. Preoperative identification is largely dependent on imaging studies, with CT imaging playing a crucial role in diagnosing and characterizing atypical hernias by delineating hernia contents, hernia orifice, and surrounding inflammatory changes [4].

The surgical management of appendix-containing hernias typically follows guidelines derived from Losanoff and Basson’s classification for Amyand’s hernia, which recommends an appendectomy along with primary hernia repair without mesh in the presence of inflammation or contamination [8]. Our surgical approach adhered to these recommendations, deliberately avoiding mesh implantation due to contamination risk. This aligns with established management strategies to prevent infectious complications in contaminated surgical fields [8].

Etiologically, the development of iliacus muscle hernias may result from congenital weaknesses or acquired defects in the muscle fascia, potentially exacerbated by factors such as chronic increases in intra-abdominal pressure, inflammation-driven erosion, or prior surgical procedures in the pelvic or retroperitoneal regions [3, 5, 6]. The retrocecal position of the appendix, as seen in our patient, may predispose it to unusual migration through fascial defects under conditions of inflammation or perforation.

Given the rarity of this presentation, awareness, and consideration of internal hernias in the differential diagnosis of acute abdominal pain with atypical imaging findings are essential. Prompt surgical intervention is critical to prevent complications such as perforation or worsening sepsis and achieving optimal patient outcomes.

## Conclusion

This case represents the first reported iliacus hernia containing an inflamed appendix, expanding the clinical spectrum of internal hernias involving the appendix. Work up with CT scan seems to be best at raising the suspicion for such pathologies. Increased awareness among surgeons of this rare entity is crucial for timely diagnosis and effective treatment.
